# Autism in Fragile X Syndrome; A Functional MRI Study of Facial Emotion-Processing

**DOI:** 10.3390/genes10121052

**Published:** 2019-12-17

**Authors:** Andrew G. McKechanie, Sonya Campbell, Sarah E. A. Eley, Andrew C. Stanfield

**Affiliations:** 1 The Patrick Wild Centre, The University of Edinburgh, Edinburgh EH10 5HF, UK; sonya.campbell@ed.ac.uk (S.C.); s.eley@ed.ac.uk (S.E.A.E.); andrew.stanfield@ed.ac.uk (A.C.S.); 2 NHS Lothian, Edinburgh EH1 3EG, UK

**Keywords:** fragile X syndrome, autism, functional imaging, emotion-processing

## Abstract

Fragile X syndrome (FXS) is the most common inherited cause of intellectual disability and autism spectrum disorder, and among those with fragile X syndrome, approximately 1/3rd meet a threshold for an autism spectrum disorder (ASD) diagnosis. Previous functional imaging studies of fragile X syndrome have typically focused on those with fragile X syndrome compared to either neurotypical or autism spectrum disorder control groups. Further, the majority of previous studies have tended to focus on those who are more intellectually able than is typical for fragile X syndrome. In this study, we examine the impact of autistic traits in individuals with fragile X syndrome on a paradigm looking at facial emotion processing. The study included 17 individuals with fragile X syndrome, of whom 10 met criteria for autism as measured by the Autism Diagnostic Observation Schedule (ADOS). Prior to the scan, participants rehearsed on a mock scanner to help acclimatize to the scanner environment and thus allow more severely affected individuals to participate. The task examined the blood-oxygen-level-dependent (BOLD) response to fearful and neutral faces taken from the Ekman faces series. Individuals in the autism group had a region of significantly reduced activity centered on the left superior temporal gyrus, compared to those with FXS alone, in response to the fearful faces. We suggest that autism in individuals with fragile X syndrome is associated with similar changes in the neurobiology of facial emotion processing as seen in idiopathic autism.

## 1. Introduction

Fragile X syndrome (FXS) is a neurodevelopmental disorder occurring in approximately 1 in 3000–4000 males and 1 in 6000–8000 females and is the leading inherited cause of intellectual disability [1]. Further, a significant proportion of individuals with FXS meet diagnostic criteria for autism, with FXS being the leading monogenic cause of autism.

It was recognized in some of the early described series of confirmed individuals with fragile X syndrome that there were higher levels of autistic traits, by way of social, communication, and sensory difficulties; than could be accounted for by level of intellectual disability alone [2,3,4,5,6,7]. In parallel, early studies in which groups with autism were screened for fragile X reported that up to 16% of autistic males had fragile X syndrome [8,9,10,11,12,13]. With autism now being more widely recognized, especially in those without an intellectual disability, these estimates are consequently lower, with fragile X syndrome accounting for approximately 0.5% of individuals with autism [14,15,16]. Despite the association between autism and fragile X syndrome, in many cases, the presentation of autism in the context of FXS differs subtly, but importantly, from the prototypical presentation in idiopathic autism [17] and it is not clear to what extent the autistic traits reported in FXS are the result of the same underlying process as those observed in idiopathic autism [18]. Whereas fragile X syndrome is a genetically-defined condition with a common phenotype, albeit with some variation in presentation, and an increased prevalence of the disorder; autism itself is a condition defined by its behavioral phenotype and with a wide variety of aetiologies, both known and unknown. Thus, as entities, they are categorically different, a theme previously investigated and discussed by Hall et al. [19]. Of note, is that discussion about whether autism in FXS is the same as idiopathic autism, is paralleled beyond FXS, with the validity of the idea of autism across the spectrum representing the same entity being questioned, particularly in the light of the revisions on ASD incorporated into DSM-5. This issue is far from new; indeed, even in the early years of autism research, Kanner was bemoaning the same issue; feeling that his idea of a relatively rare and pure entity was being challenged, noting that others appeared to wish to throw “diagnostic criteria to the winds” [20]. Notwithstanding this issue, given the increased co-occurrence of autism in FXS, the question of the nature of the overlap remains of interest.

One of the central features of autism is a difference in reciprocal social communication and interaction. What underlies this from a biological basis has been the basis of a number of theories. Whilst this likely varies across the various aetiologies of autism; given the clustering of features that define autism, we may expect some shared underlying biology. One possible contributing factor is a differential perception of facial emotional stimuli in autistic individuals, which may then contribute to differences in social understanding, communication, and interaction. Whilst the majority of studies show diminished facial emotion recognition in autistic individuals, there is significant variability in the findings; with heterogeneity in study paradigms likely to explain at least part of this [21]. The time it takes for emotion recognition may also be an important difference [22], with individuals with autism typically taking longer to recognize the emotion [23]. It should also be noted that the direction of the relationship between facial emotion recognition and the impairments in social interaction typical of autism is not entirely clear: diminished social interaction is likely to give less exposure to facial stimuli and therefore interfere with development of the associated neural circuitry; whilst primary difficulties in facial emotion recognition may make social interaction difficult [24]. Meta-analyses of emotion processing functional imaging studies in autism show recruitment of different brain regions during facial emotion recognition, with regions of both hypo- and hyper-activation seen [25,26]. Of these, the strongest and most consistent findings have been differences in activation in the fusiform face area (FFA) and temporal structures. In the FFA, typically hypoactivation is seen in individuals with autism [27,28,29,30,31]. In their review of studies reporting on FFA activation, Perlman reports that this FFA hypoactivation was seen in two-thirds of studies, with equal activation seen in the remainder [31]. With regards to the results in temporal structures, both hypo- and hyper-activation have been reported [25,28,29,32,33], largely with a focus on the superior temporal gyrus and the superior temporal sulcus.

Functional MRI imaging has been used in fragile X syndrome quite extensively to try and better understand some of the key differences across individuals in a variety of domains, including facial/emotion/gaze processing [34,35,36,37,38,39,40], auditory processing [41], cognitive functions (memory, attention, cognitive interference, equivalence processing, arithmetic processing) [42,43,44,45,46,47,48,49], and functional connectivity [50,51]. Interestingly, most of these studies have examined groups of individuals with FXS of a mean age of 18 or below. It is important to consider that development continues throughout adulthood, and differences may either emerge or diminish over time. As the field of research develops, replications of these studies, as well as our own, in older adults would help to shine a further light on the developmental trajectories and maturing brain in FXS.

The previous functional imaging studies of face and gaze processing in fragile X syndrome have produced relatively heterogeneous findings [34,35,36,37,38,39,40]. As with the autism literature, this has likely been the result of a combination of factors, including: imaging paradigm used, balance of gender, level of intellectual functioning of the FXS group, and choice of comparison group. Of particular note is that given the relationship (albeit not direct) between intelligence quotient (IQ) and fragile X mental retardation protein (FMRP) levels [52], it is likely that at least some of the variability will be explained by the wide range of group mean IQ (61–91) of the individuals in these studies, and thus the likely underlying FMRP levels. Of the 100 participants in these previous studies, there were 57 females and 43 males. Given that FXS is approximately twice as common in males as it is in females, this ratio of male to female participants, likely represents somewhat of a selection bias for females. These factors, at least in part, likely reflect the significant difficulties in recruiting and scanning individuals with more significant intellectual impairment, who are more likely to be male.

In the fragile X syndrome studies of emotion processing concerned with individuals of mean IQ <70 (i.e., considered to have an intellectual disability; a key feature of the full fragile X syndrome), the results showed decreased prefrontal activation in FXS compared to typically-developing (TD) controls [38,40]; increased left insula activation in FXS compared to TD controls [40]; left frontal gyrus hypoactivation in FXS compared to TD controls [35]; and increased activation in left hippocampus, left superior temporal gyrus, right insula, and left postcentral gyrus in FXS compared to TD and ASD controls [35]. In the study of neural habituation to faces, the FXS group showed significant sensitization and decreased habituation in cingulate gyrus, fusiform gyrus, and frontal cortex compared to IQ and autism-matched controls [34].

In this study, we aimed to further explore the relationship between autism and emotion-processing in individuals with fragile X syndrome. In particular, we were interested in whether the same patterns of differential neural activation during emotion-processing seen in individuals with idiopathic autism compared to typically-developing controls, would be seen in a group with FXS + autism, compared to individuals with FXS alone. Our hypothesis was that we would see reduced activation in the FFA and altered activity in superior temporal structures in the FXS + autism group.

## 2. Materials and Methods

### 2.1. Participant Recuritment

Initial recruitment was through the Fragile X Registry at The Patrick Wild Centre in Edinburgh. With the support of the UK-based family support charity, The Fragile X Society, the study was also advertised in their quarterly print newsletter and on their website. Subsequently, information sheets and letters of invitation were sent out to families registered with the society as being interested in research. Ethical permission for the study was granted by the National Research Ethics Service Scotland A Research Ethics Committee (reference 12-SS-0117).

### 2.2. Imaging Procedure

**Figure A1:**
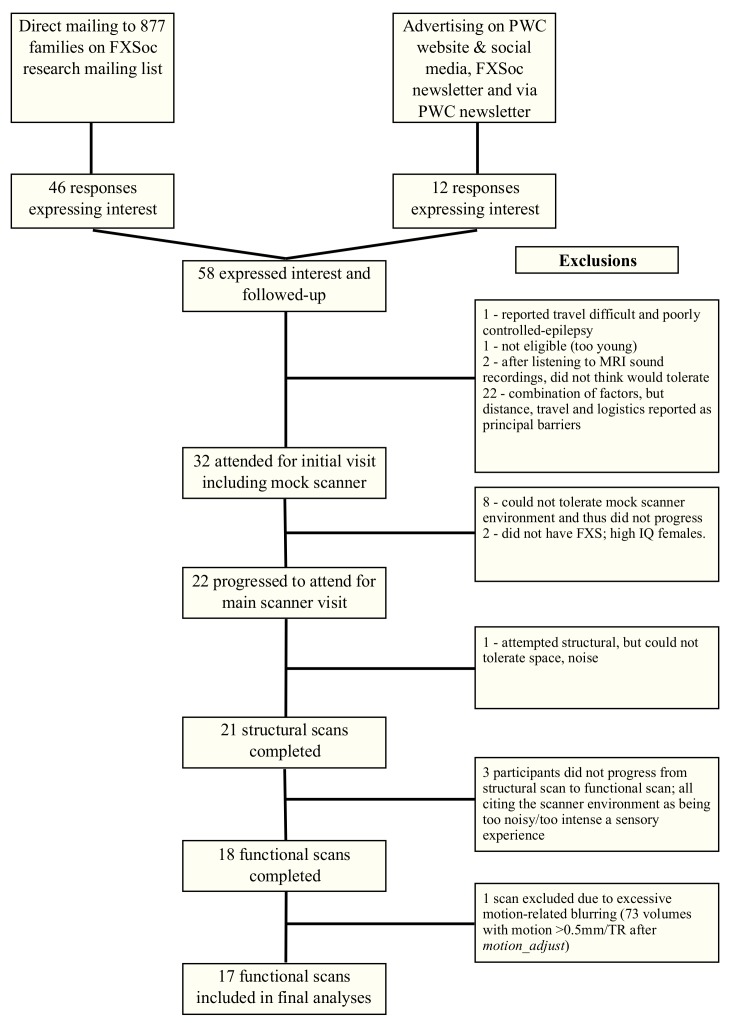
CONSORT diagram of recruitment and scanning. FXSoc—Fragile X Society.

Prior to their scan, participants were given the opportunity to rehearse the scanning procedure on two mock scanners available. The first mock scanner had been built in the Patrick Wild Centre for a previous study to facilitate desensitization to the scanning procedure, and was used extensively in this study for rehearsal and acclimatization. A further mock scanner, housed in the Clinical Research Imaging Centre (CRIC) was also used, and participants were able to rehearse on this immediately prior to their main scan. This mock scanner was a replica of the main scanner used, with the only difference being that it did not have a main coil. However, the use of earplugs, headphones, and an audio recording of the scanning sequences used in the main scanner all helped to simulate the sensory experience. Only when participants were comfortable in the mock scanner did they proceed to the main scan. Eight individuals did not successfully proceed beyond the mock scanning stage. See Figure A1 (Appendix B) for details.

### 2.3. Imaging Sequences

All scans were completed on a Siemens MAGNETOM Verio 3T scanner. For the structural imaging, using an MPRAGE sequence, a T1 structural image was obtained made up of 160 coronal slices of 1 mm slice thickness and 1 mm × 1 m × 1 mm voxels. A repetition time (TR) of 2.3 s, an echo time (TE) of 2.98 ms, flip angle of 9°, and field of view (FOV) of 256 mm were used. For the functional imaging, 159 volumes were acquired; each containing 26, interleaved, 5 mm slices of voxels 3.4 mm × 3.4 mm × 5 mm. In this case a TR of 1.56 s, a TE of 26 ms, flip angle of 66° and FOV of 220 mm were used.

The functional imaging task used was a block-design task with two main conditions, including a series of neutral faces, and a series of fearful faces, the faces being taken from the Pictures of Facial Affect series [53]. We used the fearful and neutral stimuli as differences in the processing of fearful stimuli have been shown to be particularly affected in autism [33].

A visual fixation cross was presented at the beginning and end of the sequence, as well as between the conditions of interest. In the contrasts comparing against baseline, the fixation cross was considered as the baseline condition. The complete sequence presented six blocks, each of six faces alternating between blocks of fearful or neutral faces. Within each block, each face was shown for 3.5 s with an inter-stimulus interval of 0.5 s. In between each block was an interval of 12.5 s during which a fixation cross was shown. There were two variations of the sequence, with one starting with a block of neutral faces and the other starting with a block of fearful faces; these sequences being balanced across the groups. As had been rehearsed in the mock scanners, participants were asked to depress a trigger button each time they saw an image. This was principally used as an in-scan method for ensuring participants were attending to the task, with participants needing to respond successfully on more than 80% of faces to be included in further analysis.

### 2.4. Image Processing and Analysis

#### 2.4.1. Preprocessing of fMRI Data

Images were processed and analysed using the Statistical Parametric Mapping (SPM) program (version 12, Functional Imaging Laboratory, Wellcome Trust Centre for Human Neuroimaging, University College London, London, UK; fil.ion.ucl.ac.uk/spm/) running within Matlab (R2011b (version 7.13.0.564), MathWorks, Natick, MA, USA). The ArtRepair toolbox version 5b3 [54] for SPM was used to analyze and repair motion artefacts using the single subject pipeline described by Mazaika [55]. Full details of the preprocessing pipeline are contained in Appendix A.

#### 2.4.2. Statistical Analysis of fMRI Data

For each contrast examined, a design matrix was created incorporating weightings for the neutral and fear conditions. A 128-s high-pass filter was used to remove slow signal drifts. Second-level analyses were generated using these first level contrast images for each participant to consider differences in activation, both within groups and between groups. The initial height threshold was set at *p* < 0.001 uncorrected with results considered significant at *p* < 0.05 at cluster level after family-wise error correction. Age was included as a covariate of no interest in the between-group analyses given the trend towards a significant difference between the two groups on age.

### 2.5. Measure of Cognitive Ability

The Kaufman Brief Intelligence Test (K-BIT) was used with all participants as a measure of cognitive ability. The K-BIT comprises of three sub-tests (Verbal Knowledge, Riddles, and Matrices) and takes approximately 20 min to complete [56], giving verbal, performance, and composite IQ scores.

### 2.6. Measure of Autistic Traits

The Autism Diagnostic Observation Schedule-2 (ADOS-2, henceforth, simply referred to as ‘ADOS’) was used to directly measure autistic traits. The ADOS is a semi-structured interview that uses a set of prescribed ‘presses’ to elicit, demonstrate, or create the space in which autistic features may be assessed either by the presence or absence of features that are useful in helping to establish an autism diagnosis [57]. This format allows for the assessment of autistic and associated features including 31 items across 5 domains. The 5 domains include the domains considered in autism diagnosis (social, communication, and stereotyped behaviors and restricted interests) plus the related domains of creativity and associated features.

For each participant, we used the cutoff of a combined social and communication total of ≥10 to divide the group into ‘FXS’ and ‘FXS + autism’ groups for the between-group analyses. Calibrated Severity Scores (CSS) were also calculated using the published algorithms, to provide a continuous measure of autistic traits for regression analysis. In the case of the participants who were scored on the ADOS module 4, the CSS algorithm subsequently published by Hus & Lord [58] was used.

## 3. Results

### 3.1. Feasibility of Functional Imaging in Fragile X Syndrome

Of the individuals who received the invitation to participate, or who saw the study advertisement, a total of 58 expressed interest. After discussion by telephone, 32 of these participants and their families attended an initial visit and trial on the mock scanner. Of those who did not progress from an expression of interest to a visit, a number of reasons were cited; however, for most it was the combination of all the barriers (principally distance, travel, and logistics) that were reported as being the reason not to progress. In many cases, these reasons should also be considered in the context of the participant’s and family’s uncertainty as to whether the participant would actually be able to successfully complete the scanning sequence. Of 32 participants who attended an initial visit and trial on the mock scanner, 22 attended the main scanner visit, 21 completed a structural scan, 18 completed a functional scan, and 17 were included in the analyses. Figure A1 (Appendix B) lays out the recruitment path from initial approach to completed scans.

As expected, the biggest dropout after attending an initial visit was between the mock scanner trial and the main scanner visit, emphasizing the screening role that the opportunity to rehearse on the mock scanner can provide. Whilst most of the participants had not had prior contact with the research team, a significant proportion (41%) had been involved with previous studies at The Patrick Wild Centre, and were already familiar with the staff, facilitating participation.

### 3.2. Investigating the Role of Autism in Mediating Facial Emotion Processing

To consider the relative impact of autistic traits on facial emotion processing, the data were examined in two separate ways. Firstly, by dividing the participants into two groups: those meeting the ADOS threshold for autism (social and communication total ≥10) and those not (social and communication total <10); contrasts were examined in SPM comparing the groups. Secondly, we calculated Calibrated Severity Scores (CSS) from the raw ADOS data using the published algorithms. These scores were then regressed against the contrast of interest within the autism group alone.

#### 3.2.1. Between-Group Analyses

In these analyses, we compared the response to each contrast between the groups of those with FXS alone and those with FXS + threshold autism traits on the ADOS. The makeup of the two subgroups is shown in Table 1. In general, the use of prescription medication in the participants was low, with only four participants taking regular psychoactive medication. All four participants were taking mavoglurant (AFQ056, Novartis AG, Basel, Switzerland); one in the non-autism group and three in the autism group. We consider the relatively low use of psychoactive medications in the sample to likely represent a combination of prescribing practice in the U.K. and likely a degree of selection bias—that those who were more affected and thus more likely to be on medication, were less likely to be able to participate. Further, our sample was on average older than those in most studies. As such, whilst a number of participants had been on psychoactive medications as children; they were no longer on them. Epilepsy, whilst more common in FXS, was under-represented in this sample, with no participants being treated for epilepsy.

**Figure 1:**
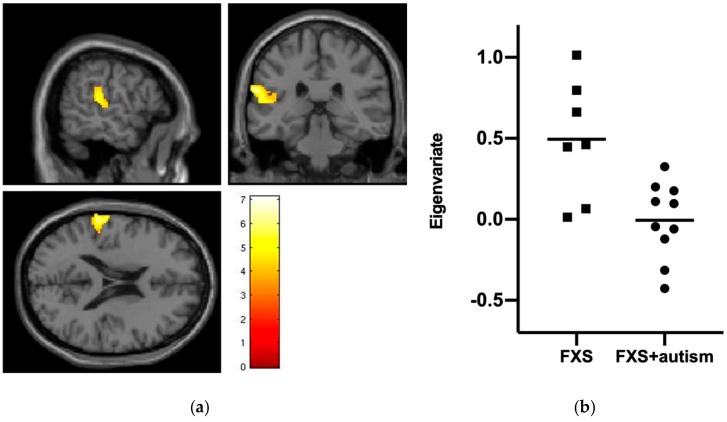
(**a**) Cluster of significantly greater brain activation in the non-autism group, compared to the autism group during the fearful faces versus baseline contrast. Region projected on the canonical single subject T1 image from SPM12. (**b**) Extracted Eigenvariate values in the two groups from the cluster shown in panel (**a**).

In the analysis comparing response to fearful faces versus baseline, there was a region of significantly different activity between the groups centered on the left superior temporal gyrus (STG) and extending to the rolandic operculum and supramarginal gyrus. Specifically, this region showed significantly greater activation in the FXS group, compared to the FXS + autism group. Co-ordinates in Montreal Neurological Institute (MNI) space for this cluster are given in Table 2. The cluster is shown in Figure 1a and the extracted values of the Eigenvariates are plotted in Figure 1b. The cluster remains significant (*p*_FWE-corr_ = 0.002; *k*_E_ = 511; Z_≡_ = 4.52; x, y, z = −64, −30, 22) when including medication use as a covariate.

There were no clusters of significant difference between the groups when considering response to neutral faces vs. baseline, or on the more subtle contrast of fearful faces vs. neutral faces. Whilst we did see the expected activation in the fusiform face area in both groups in response to the facial stimuli (both fearful and neutral), there were no between-group differences found.

#### 3.2.2. Correlation between ADOS Calibrated Severity Score and Response to Fearful and Neutral Faces

**Figure 2:**
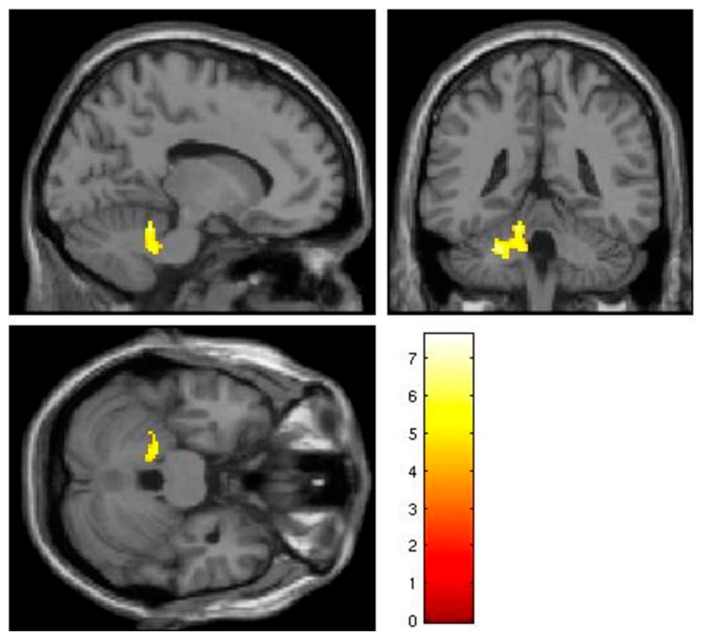
Cluster of activation significantly correlated with ADOS Calibrated Severity Score in the autism subgroup. Region projected on the canonical single subject T1 image from SPM12.

**Figure 3:**
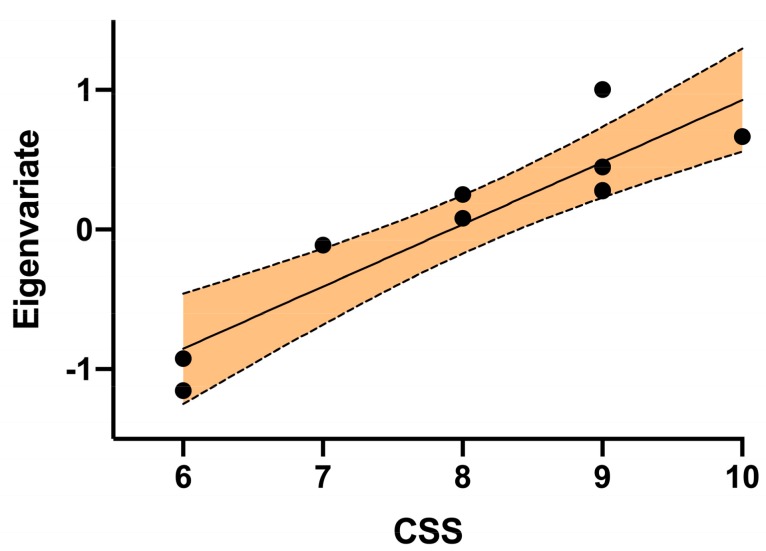
Scatter plot of the correlation between extracted Eigenvariate value and Calibrated Severity Score (CSS) from the cluster shown in Figure 2. The solid line shows the linear regression, with the dotted lines demarcating the 95% confidence bands.

In this analysis, the calculated CSS score was regressed against response to each of: all faces, neutral faces, and fearful faces in the autism subgroup. There was a cluster of positive correlation between CSS score and the response to all faces, as reported in Table 3. The region is shown in Figure 2 and Figure 3 shows the correlation between CSS and the extracted value of the cluster Eigenvariates.

While there was no significant correlation to either of the neutral > baseline or fear > baseline contrasts at a whole-brain level, a small volume correction (SVC) centered on the area of significant activation for the faces > baseline contrast was used to investigate which contrast was driving the faces > baseline result. Using this SVC, an area of significant activation was identified in the neutral > baseline contrast, whereas there was no significant activation in this area under the fear > baseline contrast. The results are shown in Table 4.

## 4. Discussion

In this study, we explored the role of autism in facial emotion processing in individuals with fragile X syndrome. Previous functional imaging studies of facial emotion processing in both autism and FXS had suggested that differences in activation in the fusiform face area and the superior temporal gyrus were the most robust findings. Interestingly, whilst we did elicit significant FFA activation at a whole group level, we did not detect any differences between the groups.

However, our finding of significantly reduced activation in the left superior temporal gyrus (STG) / superior temporal sulcus (STS) in those with FXS+ autism compared to those with FXS alone overlaps the previous findings in individuals with idiopathic autism [26]. Our result also replicates the finding of Dalton of increased activity in the same region in individuals with FXS compared to both typically-developing and autism controls [35]. Interestingly, in the FXS group reported by Dalton, none of them had a clinical diagnosis of autism, and the group had relatively low average autistic traits, as measured by the Social Communication Questionnaire (SCQ) (mean SCQ of 9). Thus, we suggest that the FXS group studied by Dalton is likely to be comparable to our non-autism FXS group. Taken all together, the result suggests that autism, whether idiopathic or associated with FXS, may be associated with the same impact on the neurological underpinnings of facial emotion processing.

Our finding in the cerebellum of a correlation between activation to neutral faces and CSS scores is interesting in that the findings for the role of the cerebellum in social processing in autism have generally been that cerebellar activation is diminished in individuals with autism compared to typically-developing controls [59,60,61,62]. However, in their meta-analysis of 350 fMRI studies examining the role of the cerebellum and social cognition, Van Overwalle et al. suggest that cerebellar activity may actually increase when the level of abstraction in the task increases, and with it the demand on executive resource [63]. If neutral faces are considered to be more ambiguous and thus may appear more abstract than overtly emotional (in this case, fearful) faces, then this may be an explanation for the finding. However, given that this finding is largely in contrast to the literature, we think it only appropriate to be circumspect with regard to this finding. Further, given its level of significance at a whole brain level, we can be less confident in it. Indeed, correcting for multiple comparisons, the result would no longer be significant.

In the between-group comparisons, we were looking at three main contrasts: response to neutral faces versus baseline, response to fearful faces versus baseline, and response to fearful faces versus neutral faces. Although the only significant differences that we noted were in the fearful versus baseline contrast, as noted in the results, we did not find any group differences on the fearful versus neutral contrasts. We therefore cannot specifically ascribe our findings to the processing of emotional content per se and it is possible that they relate to more general face processing differences.

Beyond the findings from our imaging analysis, we also describe the methods used in successfully imaging a cohort of individuals with fragile X syndrome who were more intellectually impaired than many participants in prior studies. The mean full scale IQ of individuals in this study was 60.9, while images were successfully acquired in individuals with IQ as low as 40. Using these methods, we have, therefore, shown that it is possible to successfully image people with FXS and significant ID. We hope that the descriptions of methods used and the description of where participants dropped out from the study will be of use to other researchers in planning similar studies.

### 4.1. Limitations

#### 4.1.1. Participants

The study has a number of limitations. Firstly, as with many of the previous imaging studies in fragile X syndrome, larger numbers would have provided more power to detect more subtle group differences. Nonetheless, this is still one of the larger functional imaging study of males with FXS and we hope that it can add to the literature. Acknowledging the difficulties in recruiting the most severely affected individuals, there was likely a degree of selection bias; with a number of potential participants either not responding to the invitations, or dropping out once they had tried the mock scanner. In terms of comparison or control groups, previous studies in FXS have taken a variety of approaches: typically-developing controls, ASD controls, typically-developing developmental age-matched controls or developmental delay controls. In this study, we were interested in the effect of autism on individuals with FXS and thus chose to compare two groups of individuals with FXS; one with high autistic features, and one without. It would have been interesting to also include further comparison groups; however, that was beyond the scope of this study, although is a focus of currently ongoing work.

#### 4.1.2. Measures

The ADOS, whilst commonly used, is not validated for use in adults with such significant intellectual disability; although the bimodal distribution of both ADOS total and CSS scores gives a degree of confidence that our groups represented individuals with significant differences in social communication and interaction. Had we chosen the lower threshold of ≥7 for autism spectrum disorder as published in the ADOS, we would still have had groups of the same makeup. Further, the ADOS was completed by two researchers (A.G.M. (consultant psychiatrist) & S.C. (clinical & research psychologist)) who are trained as research-reliable ADOS users, adding to the degree of reliability that can be afforded to the findings. A full clinical workup considering autism diagnosis, would of course have been preferable, however, was not feasible within the constraints of the study.

#### 4.1.3. fMRI Paradigm and Acquisition

During the acquisition of the imaging data, we used a trigger for the participants to indicate when they had seen a face, with participants needing to respond successfully on more than 80% of faces to be included in further analysis. However, our scanning facility unfortunately did not have in-scanner eye-tracking available, and as such we do not know for how long, or with what pattern, the participants visually attended to the stimuli. Given that both FXS and autism are associated with gaze aversion, and that there may be a group difference on gaze, we cannot be confident that our results do not represent differences in gaze, either instead of, or as well as, differences in underlying neural processing. The use of a fixation cross as baseline also means that we cannot attribute our findings specifically to face processing, as opposed to more general visual perceptual differences between the groups. Future research should include eye-tracking combined with a higher-level baseline, such as a scrambled face, to address this.

## 5. Conclusions

In this study, we have shown that autism in individuals with fragile X syndrome is associated with the same reduction in activation in the left superior temporal gyrus, as is seen in individuals with both idiopathic autism, compared to typically-developing controls; and in idiopathic autism, compared to non-autistic individuals with FXS. This supports the idea that autism in FXS may, at least in part, represent a good model for autism more broadly.

## Figures and Tables

**Table 1:** Baseline details of participants included in the imaging analyses.

	Autism Group	Non-Autism Group
N	10	7
Male: female	8:2	5:2
Age	18 (6.2)	27 (11.7)
Full-scale IQ	59 (8.9)	63 (14.2)
Verbal IQ	69 (11.9)	71 (14.2)
Performance IQ	58 (11.1)	60 (14.4)
ADOS Total	15 (10–20)	2 (0–5)
ADOS CSS	8 (6–10)	2 (1–2)

Results show group means (s.d.) for age and IQ and median (range) for the ADOS scores. The groups were not significantly different on gender (*p* = 0.682), age (*p* = 0.092), full-scale IQ (*p* = 0.528), verbal IQ (*p* = 0.695) or performance IQ (*p* = 0.704). ADOS, Autism Diagnostic Observation Schedule; CSS, Calibrated Severity Score.

**Table 2:** Region of significantly different response to fearful faces between FXS and FXS + autism groups.

Cluster	*p*_FWE-corr_	*k*_E_	Z_≡_	x	y	z
Left superior temporal gyrus	0.001	570	4.45	−64	−30	22

Significance given as cluster-level, familywise-error corrected value. x, y, z co-ordinates given in MNI space.

**Table 3:** Region of significant correlation between ADOS Calibrated Severity Score and response to all facial stimuli.

Cluster	*p*_FWE-corr_	*k*_E_	Z_≡_	x	y	z
Left cerebellum, anterior lobe, lobules IV/V	0.029	198	4.01	−24	−42	−34

Significance given as cluster-level, familywise-error corrected value. x, y, z co-ordinates given in MNI space.

**Table 4:** Region of significant correlation between ADOS Calibrated Severity Score and response to neutral facial stimuli using the small volume correction described.

Cluster	*p*_FWE-corr_	*k*_E_	Z_≡_	x	y	z
Left cerebellum, anterior lobe, lobules IV/V	0.006	27	3.70	−20	−38	−30

Significance given as cluster-level familywise-error corrected value. x, y, z co-ordinates given in MNI space.

## Appendix A

Images were analyzed using the Statistical Parametric Mapping (SPM) program (version 12, Functional Imaging Laboratory, Wellcome Trust Centre for Human Neuroimaging, University College London, London, UK; fil.ion.ucl.ac.uk/spm/) running within Matlab (R2011b (version 7.13.0.564), MathWorks, Natick, MA, USA). Data were initially reconstructed using the DICOM Import function within SPM for further processing within SPM.

Prior to pre-processing, the first seven volumes of the functional scans were discarded to reduce the impact of T1 equilibrium effects. Images were initially realigned to the mean EPI image using the Realign (realign and unwarp) module of SPM. The T1 structural image was co-registered to the mean EPI image. The images were realigned, before being smoothed with a 4 mm FWHM Gaussian smoothing kernel. The ArtRepair toolbox version 5b3 [54] for SPM was used to first examine and then repair the volumes, using the *art_motionregress* and *art_global* modules of the single subject pipeline described by Mazaika [55] The *art_motionregress* algorithm of ArtRepair was used as an alternative to adding the movement parameters in the design matrix, as the motion regressors have been described as not being sufficiently accurate to account for the relatively larger movements of clinical subjects [64]. The images were subsequently analyzed and repaired using the *art_global* module in ArtRepair. In this step, the volumes were examined for fast head movements, and volumes with movement of >0.5 mm/TR were interpolated with the nearest usable volumes. 10/17 scans had to be repaired in this way. Where a scan had >20% of volumes with >0.5 mm/TR movement, the scan was excluded. Only 1 scan had to be excluded for this reason. The T1 structural image was segmented before both structural and functional images were normalized using normalization parameters arising from the T1 image segmentation. Finally, a 7 mm kernel was used in a second smoothing step; the combination of 4 mm and 7 mm smoothing being approximately equivalent to the commonly-used single 8 mm kernel [64].
